# Immunogenicity of COVID-19 Vaccination in Melanoma Patients under Immune Checkpoint Blockade

**DOI:** 10.1159/000524894

**Published:** 2022-05-24

**Authors:** Jacqueline Niewolik, Marie Mikuteit, Anne Cossmann, Kai Vahldiek, Ralf Gutzmer, Frank Müller, Dominik Schröder, Stephanie Heinemann, Georg M.N. Behrens, Alexandra Dopfer-Jablonka, Sandra Steffens, Imke Grimmelmann

**Affiliations:** ^a^Department of Rheumatology and Immunology, Hanover Medical School, Hannover, Germany; ^b^Dean's Office − Curriculum Development, Hannover Medical School, Hannover, Germany; ^c^CiiM − Center for Individualized Infection Medicine, Hannover, Germany; ^d^Institute for Information Engineering, Ostfalia University of Applied Sciences, Wolfenbüttel, Germany; ^e^Department of Dermatology, Johannes Wesling Medical Center Minden, Ruhr University Bochum, Minden, Germany; ^f^Department of General Practice, University Medical Center Göttingen, Göttingen, Germany; ^g^German Center for Infection Research (DZIF), partner site Hannover-Braunschweig, Hannover-Braunschweig, Germany; ^h^Department of Dermatology and Allergy, Skin Cancer Center Hannover, Hanover Medical School, Hannover, Germany

**Keywords:** COVID-19 vaccination, Advanced melanoma, Immune checkpoint inhibitors, Immunogenicity, SARS-CoV-2

## Abstract

**Background:**

Immunogenicity of SARS-CoV-2 vaccines is modestly impaired in cancer patients due to a generally weakened immune system. Immune checkpoint inhibitors (ICI) are expected to enhance immune response. This has already been described to be the case in influenza vaccines, and first data about COVID-19 vaccines show a trend in this direction.

**Aim:**

We aimed to investigate the immune response of patients with melanoma under ICI therapy after COVID-19 vaccination.

**Patients and Methods:**

In the Skin Cancer Center Hanover (Germany), we recruited 60 patients with advanced melanoma who either received ICI therapy during or before the vaccination period. Serological blood analysis was performed using quantitative ELISA for Anti-SARS-CoV-2 spike protein 1 IgG antibodies.

**Results:**

We did not observe an enhanced humoral immune response in patients under active or past ICI therapy after COVID-19 vaccination. Nevertheless, there is a tendency of higher antibody levels when ICI therapy was received within the last 6 months before vaccination. Subgroup analysis revealed that patients in our study population under ongoing targeted therapy during vaccination period had significantly higher median antibody levels than patients without any active antitumor treatment.

**Conclusion:**

Melanoma patients under ICI therapy show comparable antibody response after SARS-CoV-2 vaccination to healthy health care professionals. This finding is independent of the timing of ICI therapy.

## Introduction

Due to their weakened immune system, cancer patients are generally considered at higher risk for Coronavirus Disease 2019 (COVID-19) [1, 2]. Many vaccination programs have thus included cancer patients very early. There is quite a variance in the effectiveness of COVID-19 vaccination within this group of cancer patients [3]. A recent systematic review shows varying seroconversion rates between 47.5 and 100% after two doses of vector- or mRNA-based vaccines for patients with solid tumors [4].

Among patients with metastatic melanoma under therapy with immune checkpoint inhibitors (ICI), very few cases of severe COVID-19 were described [5]. Before the implication of adjuvant therapies like ICI and targeted therapy (TT), melanoma was one of the malignancies with the highest potential of dissemination and very poor prognosis with 5 year-survival rate between 5 and 19.0% [6]. Therapy of metastatic melanoma with ipilimumab, nivolumab, and pembrolizumab now shows a 12-month rate of recurrence-free survival of 61.6%, 72.3%, and 75.4%, respectively [7, 8].

Cancer patients under ICI can hardly be generalized under the overarching group of cancer patients with weakened immune response. Through blockade of the T-cell inhibiting PD-L1 and CTLA-4, ICI are enhancing the immune responses against cancer. This may lead to higher levels of anti-spike IgG compared to patients under chemotherapy or TT [9]. In addition, studies examining the seroprotection against influenza virus could also show significantly higher seroconversion rates in subgroups undergoing ICI in comparison to those undergoing cytotoxic chemotherapy [10].

Therefore, the aim of this study was to investigate the immune response in patients with metastatic melanoma under immunotherapy by measuring anti-Spike-1 IgG antibodies after COVID-19 vaccination. To gain insight into the long-term effects on immunogenicity, we also included patients who have previously undergone ICI therapy (>6 months).

## Patients and Methods

### Recruitment and Eligibility Criteria

In a prospective cohort study, we included from May to October 2021 adult stage III or IV melanoma patients (AJCC melanoma staging system, [11]) that were treated in the Skin Cancer Center Hannover of the Department of Dermatology at Hanover Medical School (MHH). Inclusion criteria were a previous or active treatment with ICI such as PD-1 inhibitors nivolumab, pembrolizumab, CTLA-4 inhibitor ipilimumab, or a combination thereof and a completed COVID-19 vaccination schedule. Serological blood analysis was done within a time span from 20 to 218 days after completing COVID-19 vaccination. Exclusion criteria were refusal or inability to give informed consent and contraindication to blood testing. The study group was part of a larger protocol, investigating the immune response after vaccination in immunocompromised and elderly persons. Written informed consent was obtained from each patient before enrollment. The study was approved by the Ethics Committee of Hannover Medical School (Approval No. 9948 BO K 2021) and registered in the German Clinical Trial Registry (DRKS00023972).

### Samples, Measurements, and Analysis

For serological testing, a semiquantitative ELISA for SARS-CoV-2 spike protein 1 IgG was used (Euroimmun, Lübeck, Germany − according to manufacturer's instructions (dilution up to 1:2,000). According to the WHO International Standard for COVID-19 serological test, the results (here given in RU/mL) can be converted into binding antibody units (mL) with a conversion factor of 3.2 [12]. EDTA blood samples were either collected during appointments in our outpatient clinics, through a local health care provider, or self-sampling. Additional clinical data, including immunosuppressive comedication (ICo), tumor staging, and the occurrence of immune-related adverse events (irAE) was collected by interviews, by questionnaires, and by chart review of electronic medical records.

Median antibody levels within our study population were compared according to the therapies received during vaccination period and according to the timing of ICI therapy in relation to vaccination period. Vaccination period was defined as the period between the first and 2 weeks after the final dose of vaccination.

### Statistical Analysis

Data were stored pseudonymized using Microsoft Excel 2019, version 2010 (Microsoft Corporation, Redmond, WA, USA) password secured on the internal server of Hanover Medical School. Statistical analysis was performed using IBM SPSS Statistics for Windows, version 27 (IBM Corp., Armonk, NY, USA) and open-source statistics software R. Data on patient parameters were described as proportions with percentages or as medians with interquartile range (IQR). The continuous variable antibody level was analyzed using Wilcoxon test. A *p* value <0.05 was considered statistically significant.

## Results

### Study Population

Overall, 67 patients met the inclusion criteria and were included, 7 patients were lost to follow-up after the completed vaccination. Twenty-two (36.7%) were female and 38 (63.3%) were male. Median age was 61.0 years. The study population included 25 (41.7%) stage III A–D and 35 (58.3%) stage IV M1a-M1d-advanced melanoma patients. The majority of patients (*n* = 51, 85%) were vaccinated with two doses of mRNA vaccines (BNT162b6 or mRNA-1273). Detailed patients' characteristics are presented in Table 1.

During vaccination period, 16 (26.7%) patients received ICI therapy, 10 (16.7%) patients TT (BRAF inhibitors and MEK inhibitors), 1 chemotherapy, and 33 (55.0%) had no ongoing tumor therapy (NTT). Of those patients who weren't currently under ICI, 14 (23.3%) patients had received their last dose of ICI within the last 6 months and 32 (53.3%) patients more than 6 months before vaccination. ICo was taken by 19 (31.7%) patients during the vaccination period due to irAE or autoimmune disease. The median antibody level of the whole study population was 205.5 RU/mL (IQR: 83.3–665.1 RU/mL). The median time span between completed vaccination and blood analysis was 89.5 days (IQR: 67.0–107.0 days).

### ICI, Short- and Long-Term Effects on Immunogenicity

**Fig. 1:**
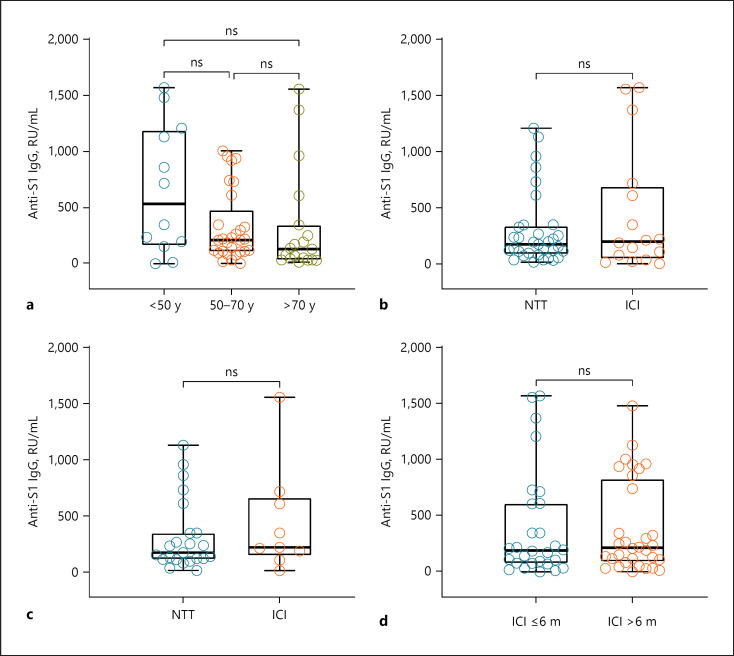
Median antibody levels (**a**) of all patients according to age group (*n* = 60) with 12 patients under 50 years, 29 patients between 50 and 70 years, and 19 patients older than 70 years; according to ongoing antitumor therapy in vaccination period (*n* = 49) with 33 patients under NTT and 16 patients under ICI therapy (**b**); in patients without ICo according to ongoing antitumor therapy in vaccination period (*n* = 31) with 23 patients under NTT and 9 patients under ICI therapy (**c**); according to timing of ICI therapy of patients (*n* = 60) with 28 patients who received ICI within the last 6 months, and 16 patients who received ICI more than 6 months ago (**d**). NTT, no ongoing tumor therapy; ICI, immune checkpoint inhibitors; TT, targeted therapy; ns, nonsignificant difference; m, months; y, years.

There was no difference between patients' median antibody levels that were under ongoing ICI therapy during the vaccination period (*n* = 16) and those who received NTT during vaccination period (*n* = 33; Fig. 1b). There was still no difference when only regarding patients without ICo (Fig. 1c).

When regarding the long-term effects of ICI therapy, no significant difference in median antibody levels between recent or past ICI therapy could be shown (Fig. 1d). Excluding patients receiving ICo and/or TT showed no significant difference but a tendency with higher median antibodies in patients who received ICI within the last 6 months before vaccination (*n* = 16, 216.0 RU/mL) than in those who had received it before more than 6 months before vaccination (*n* = 16, 147.0 RU/mL).

Median antibody levels were highest in the youngest age group of patients below 50 years (533.8/IQR 177.1–1,169.7 RU/mL) and lowest in the patients above 70 years (131.9/IQR 42.0–299.9 RU/mL; Fig. 1a). However, within our study population, age could not be identified as a significant risk factor for lower antibody levels. Patients who were analyzed within a time span of 50 days after the vaccination showed higher median antibody levels (725.1/IQR 348.7–1,208.2 RU/mL) than patients who were analyzed 51–100 days (199.8/IQR 73.6–744.1 RU/mL) or more than 100 days after vaccination (121.6/IQR 79.9–221.0 RU/mL). The median antibody levels between PD-1 inhibitor monotherapy versus ipilimumab monotherapy versus a combination of both did not vary significantly (*p* = 0.58). The median antibody levels do not vary significantly regarding vaccine type (*p* = 0.54).

### ICo and TT

**Fig. 2:**
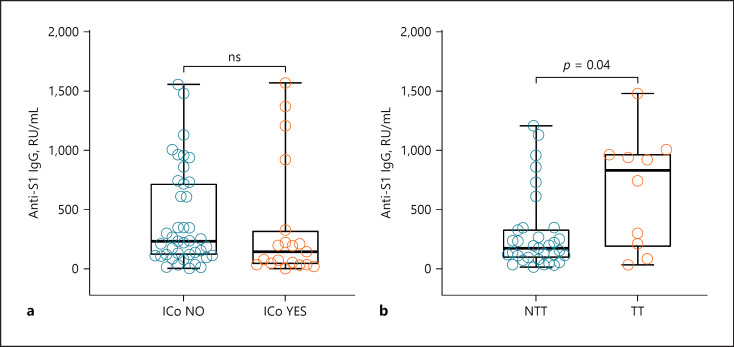
Median antibody levels according to (**a**) whether ICo was received during vaccination period (*n* = 19) or not (*n* = 41); regarding NTT (*n* = 33) versus TT (*N* = 10) received during vaccination period (**b**). ICo NO, no immunosuppressive comedication during vaccination period; ICo YES, received immunosuppressive comedication during vaccination period; NTT, no ongoing tumor therapy; TT, targeted therapy; ns, nonsignificant difference.

There was a substantial but statistically nonsignificant difference (*p* value = 0.17, Wilcoxon Test) in median antibody levels with lower levels in the 19 patients under ICo during vaccination period (146.1 RU/mL) compared to those who did not receive any ICo (233.7 RU/mL, Fig. 2a). When comparing the immunosuppressive medication in detail, patients taking more than 20 mg/day prednisone equivalent, rituximab, or MTX medication had the lowest antibody levels. Therapy characteristics and median antibody levels are shown in Table 2.

Subgroup analysis revealed that the 10 patients under ongoing TT during vaccination period had higher median antibody levels compared to the 16 patients under NTT in vaccination period (Fig. 2b, *p* value = 0.04). The 10 patients receiving TT during vaccination period were part of the group who received ICI therapy more than 6 months before vaccination and showed a similar distribution of age (median age: 59.0 years/IQR 54.5–67.5 years) and timespan after completed vaccination (90 days/IQR 85.0–104.5 days) compared to the whole study population.

## Discussion

To our knowledge, this is the largest study so far investigating the effect of ICI therapy on the immunogenicity of COVID-19 vaccines in patients with malignant melanoma. Taking under consideration the decline of antibodies after vaccination, the median antibody level of our study population (205.5 RU/mL) is comparable with median antibody levels found in a group of 57 healthy health care professionals shown in a recently published study by our group [14].

There was neither a significant difference in median antibody level between patients undergoing active ICI therapy during vaccination period and those who did not nor when considering the long-term effect of ICI therapy. However, when considering patients without ICo or TT during vaccination period, a tendency towards a positive effect of ICI taken within 6 months before vaccination on the immunogenicity was shown in this study cohort. These results are quite more ambiguous than what was expected due to the immune-stimulating mechanism caused by ICI therapy and as suggested by other studies [9]. Di Giacomo et al. [9] showed that after only one dose of vaccine, patients receiving ICI therapy develop significantly higher levels of antibodies than patients receiving chemotherapy. Similar differences were described for influenza vaccination [10].

A risk factor for lower antibody titers after SARS-CoV-2 vaccination seems to be ICo [15]. ICI are known to trigger irAE which are then, as 31.7% of our population, often treated with ICo such as steroids. Compared to the group of patients who did not have to take immunosuppressants, the antibody titers of the patients with ICo were lower, as expected. However, we could not see a significant difference between patients under ICo during vaccination period compared to those without. This might be due to the small number of patients.

While the immunosuppressive effects of chemotherapy, corticosteroids, and rituximab are plausible, a previous study has reported a lower seroconversion rate of patients treated with TT (CDK 4/6 inhibitors, PARP inhibitors, tyrosine kinase inhibitors) [16]. Interestingly, in our study, patients undergoing TT (BRAF- and MEK inhibitors) showed higher median antibody levels. This effect could not be explained by uneven age distribution or a different distribution of time span between analysis and vaccination.

An Israeli study published by Ligumsky et al. [17] in 2021 compared median antibody levels in 326 patients receiving different anticancer treatments. The sample size of patients only receiving immunotherapy (*n* = 55) was comparable to our study. Similar to our study, they showed a tendency for higher median antibody levels in patients under TT than in patients under active ICI. In their comparison to patients under chemotherapy, there were significantly higher levels in patients under TT. Unlike our study, the Israeli study did not investigate the long-term effects of ICI therapy but compared only different active therapies [17]. One explanation for this inconsistent finding could be the differences in targeted therapies used in the respective studies.

Cancer patients under immune checkpoint blockade are not specifically represented in the big group of cancer patients with weakened immune response. Through blockade of the T-cell inhibiting PD-L1 and CTLA-4, ICI as nivolumab, pembrolizumab, and ipilimumab are enhancing the immune responses against cancer. Hypothetically, this mechanism should also enhance the immune response to vaccinations as the COVID-19 vaccines.

Furthermore, higher age was a risk factor for lower antibody levels in this study population. This finding is congruent with studies on the effect of age on the number of antibodies after COVID-19 vaccination. Even if seroconversion rate for the general population including the elderly is 100%, studies have pointed at age as a risk factor for faster antibody decline after SARS-CoV-2 vaccination [18].

Our study has several limitations. First, the time span between vaccination and antibody analysis was very variable. Second, a matched control group is lacking thus relying on the comparison to our previously published findings of a large group of health care professionals [14]. The inherent small cohort and skewness of the data limited the statistical analysis. Finally, the neutralizing capacity of the antibodies or the cellular immune response after vaccination was not analyzed.

In conclusion, melanoma patients under ICI therapy show sufficient antibody building after SARS-CoV-2 vaccination. An enhanced immune response to the COVID-19 vaccination could not be shown between patients previously or actively undergoing ICI therapy. Interestingly, patients undergoing TT with BRAF- and MEK inhibitors showed higher median antibody levels. When evaluating the best timing for vaccination, e.g., the third or further COVID-19 boosters in patients with ICI treatment, age, and the effect of immunosuppressive medication have to be considered. The role of TT and ICo regarding immunogenicity of COVID-19 vaccination in melanoma patients needs further research.

## Statement of Ethics

The study was approved by the Ethics Committee of Hannover Medical School (Approval No. 9948 BO K 2021) and registered in the German Clinical Trial Registry (DRKS00023972). Informed written consent was obtained from all individual participants included in the study. Details that disclose the identity of the subjects under study were omitted.

## Conflict of Interest Statement

Ralf Gutzmer declares speaker honoraria from Roche, BMS, MSD, Novartis, Amgen, Merck Serono, Almirall Hermal, SUN, Sanofi, and Pierre-Fabre. Advisory board honoraria from BMS, Roche, Novartis, Almirall Hermal, MSD, Amgen, SUN, Sanofi, Pierre-Fabre, 4SC, Bayer, MerckSerono, Pfizer, and Immunocore; and research grants from Novartis, Pfizer, Johnson & Johnson, Amgen, Merck-Serono, SUN Pharma, and Sanofi; and travel/meeting support from Roche, BMS, SUN, Merck-Serono, and Pierre-Fabre. Imke Grimmelmann declares speakers and advisory board honoraria from Almirall Hermal, Bristol Myers Squibb, Merck Sharp & Dome, Novartis, Pierre Fabre, Sanofi Genzyme, and SUN Pharma. All other authors declare that they have no competing interests.

## Funding Sources

The study was part of the DEFEAT Corona project, funded by the European Regional Development Fund (EFRE, Funding No. ZW7-85152953 and ZW7-85151373). The funding source had no influence on the design or execution of the study, data analysis, or interpretation.

## Author Contributions

Study conception and design: Imke Grimmelmann, Sandra Steffens, Alexandra Dopfer-Jablonka, and Jacqueline Niewolik; acquisition of data: Jacqueline Niewolik and Marie Mikuteit; experimental laboratory work: Anne Cossmann; analysis and interpretation of data: Jacqueline Niewolik, Kai Vahldiek, Georg M.N. Behrens, Imke Grimmelmann, Sandra Steffens, and Dominik Schröder; drafting of manuscript: Jacqueline Niewolik; critical revision: Sandra Steffens, Imke Grimmelmann, Ralf Gutzmer, Frank Müller, Georg M.N. Behrens, and Stephanie Heinemann.

## Data Availability Statement

The datasets generated during and/or analyzed during the current study are available from the corresponding author on reasonable request.

## Figures and Tables

**Table 1:** Patients' characteristics

Variable	Frequencies, *n* (%)
Age, years	
<50	12 (20.0)
50–70	29 (48.3)
≥71	19 (31.7)
Vaccination	
mRNA vaccines (BNT162b6 or mRNA-1273), *n* (%)	51 (85.0)
2× AZD1222 (Astra Zeneca), *n* (%)	5 (8.3)
Other combinations^1^	4 (6.7)
Tumor therapy during vaccination period	
ICI	16 (26.7)
TT	10 (16.7)
Chemotherapy	1 (1.7)
NTT	33 (55.0)
Timing of ICI therapy	
ICI within 6 months before 1st dose of vaccine	28 (46.7)
ICI more than 6 months before 1st dose of vaccine	32 (53.3)
Details of ICI therapy, *n* (%)	
Mono PD-1	42 (70.0)
Mono ipilimumab	4 (6.7)
PD-1 + ipilimumab	14 (23.3)
Best response to ICI therapy^2^	
No evidence of disease/complete remission	27 (45.0)
Stable disease/mixed response/progressive disease	30 (50.0)
Unknown	3 (5.0)
irAE,^3^*n* (%)	42 (70.0)
No irAE, *n* (%)	18 (30.0)
ICo in vaccination period	19 (31.7)
Steroids ≤5 mg/day^4^	5 (8.3)
Steroids >5 mg to 20 mg/day^4^	5 (8.3)
Steroids >20 mg/day^4^	6 (10.0)
Other immunosuppression^5^	3 (5.0)
No immunosuppression	41 (68.3)

1 One patient with Ad26.COV2.S, 2 patients with 1× AZD1222 + 1× mRNA-based vaccine, 1 patient with a SARS-CoV-2 infection and 1× mRNA-based vaccine.

2 iRECIST guidelines [13].

3 Autoimmune-related adverse events (defined by the National Cancer Institute's Common Terminology Criteria CTCAE).

4 Equivalent prednisone dose per day.

5 Other immunosuppressive medication, 2 patients with MTX and one with rituximab.

**Table 2:** ICo and median antibody levels

	*n* (%)	Antibody level median in RU/mL (IQR)
Total group	60 (100)	
Immunosuppression in vaccination period	19 (31.7)	146.1 (41.6–276.0)
No immunosuppression	41 (68.3)	233.7 (113.5–717.7)
Steroids^1^ ≤5	5 (8.3)	199.8 (194.8–212.5)
Steroids >5–20 mg	5 (8.3)	223.6 (47.3–921.7)
Steroids >20 mg/day	6 (10.0)	109.8 (33.2–1,208.2)
Other immunosuppression^2^	3 (5.0)	35.9 (18.9–45.6)

1 Equivalent prednisone dose per day.

2 Other immunosuppressive medication: 2 patients with MTX and 1 with rituximab.
